# The Sufferings of Christ

**Published:** 1887-11-26

**Authors:** 


### WORDS OF CONSOLATION.

[Communications for this column should be addressed "Chaplain," care of the Editor of THE HOSPITAL, who will gratefully welcome any suggestions or inquiries from matrons, nurses, and the staff generally.]

### LX.—The Sufferings of Christ.

(*For Reading to the Sick.*)

"His visage was so marred more than any man, and his form more than the sons of men." (Isaiah lii. 14.) Let us consider some reasons for the marring of His face and the disfigurement of His countenance. When we behold Him with His face all bruised and torn and discoloured, His forehead and cheeks streaming down with blood; verily the prophet's words have their most literal fulfilment. "There is no beauty that we should desire him." (Isaiah liii. 2.) Did you ever notice the reason for the blood flowing so profusely, as we are led to think it did, from His face. The crown of thorns was beaten down on to His head. They smote Him with the reed on the head just after the crown had been placed there. Then seems to have been the time when He endured the worst in the way of scorn and contempt. He looked for some one to have pity on Him; but there was no man to care. The utmost scorn and ridicule seem connected with this marring of His sacred countenance. We do not know what our Lord's personal appearance was like; but we do know that at this period of the Passion He was most cruelly disfigured. Was this permitted in order to atone for sins of pride and vanity founded upon personal appearance—the vanity connected with human beauty real or fancied? Ah! how common and how depraving a sin this is. A face once flattered, then thought too much of, then idolised and worshipped instead of its author. Does that find a much needed atonement in this episode of the Passion? Look of the ways (their name is legion) whereby the human countenance is degraded, or, as vanity would have it, made attractive. Consider the weight of sin contracted in this direction, whether by the silly multiplication of ornaments, the contrivances of fashion, or by false colouring—many of them bestowed with such diligence upon the head—whereby we are specially marked out amongst His creatures as in the image and glory of God, and you may see a reason indeed for the marring of His visage and for the crown of thorns. You who are sick and care little for these things now—if you ever did—you can see more clearly than some others how utterly contemptible is all vanity and conceit founded on appearances; and yet how much it takes to convince people of this kind of folly! Does not the love of the Saviour make the strongest possible appeal to the vain-glorious, from this depth of His degradation, to put aside their sin, and learn of Him to be meek and lowly? Otherwise, what if these lovers of fashion, so fond of themselves, do not repent? What if such a sacrifice for pride supplies no motive for the practice of humility? Does the Passion here throw any light, by way of warning, into the dread chambers of hell? He hid not His face from shame. He became, for our sakes, the very scorn of men and the outcast of the people. Surely the proper retribution will be this: "Some will awake to shame and everlasting contempt" (Dan. xii. 2). Do we not see this feature of retribution already manifested? Who bring the most ridicule and contempt upon themselves, even now, but the vain and the self-admirers? Whom do we secretly visit with the utmost scorn but those who strive the hardest to make a reputation, to attract notice—in a word, whose aim is to crown themselves?

